# Objective and Subjective Auditory Effects of Traumatic Brain Injury and Blast Exposure in Service Members and Veterans

**DOI:** 10.3389/fneur.2020.00613

**Published:** 2020-07-03

**Authors:** Stefanie E. Kuchinsky, Megan M. Eitel, Rael T. Lange, Louis M. French, Tracey A. Brickell, Sara M. Lippa, Douglas S. Brungart

**Affiliations:** ^1^Walter Reed National Military Medical Center, Bethesda, MD, United States; ^2^Defense and Veterans Brain Injury Center, Silver Spring, MD, United States; ^3^Henry M. Jackson Foundation for the Advancement of Military Medicine, Bethesda, MD, United States; ^4^National Intrepid Center of Excellence, Bethesda, MD, United States; ^5^Department of Psychiatry, University of British Columbia, Vancouver, BC, Canada; ^6^General Dynamics Information Technology, Falls Church, VA, United States; ^7^Department of Psychiatry, Uniformed Services University of the Health Sciences, Bethesda, MD, United States

**Keywords:** speech perception, hearing, tinnitus, traumatic brain injury, blast exposure, service members and veterans

## Abstract

Service members and veterans (SMVs) with a history of traumatic brain injury (TBI) or blast-related injury often report difficulties understanding speech in complex environments that are not captured by clinical tests of auditory function. Little is currently known about the relative contribution of other auditory, cognitive, and symptomological factors to these communication challenges. This study evaluated the influence of these factors on subjective and objective measures of hearing difficulties in SMVs with and without a history of TBI or blast exposure. Analyses included 212 U.S. SMVs who completed auditory and cognitive batteries and surveys of hearing and other symptoms as part of a larger longitudinal study of TBI. Objective speech recognition performance was predicted by TBI status, while subjective hearing complaints were predicted by blast exposure. Bothersome tinnitus was associated with a history of more severe TBI. Speech recognition performance deficits and tinnitus complaints were also associated with poorer cognitive function. Hearing complaints were predicted by high frequency hearing loss and reports of more severe PTSD symptoms. These results suggest that SMVs with a history of blast exposure and/or TBI experience communication deficits that go beyond what would be expected based on standard audiometric assessments of their injuries.

## Introduction

Traumatic brain injury (TBI) is a common injury among military service members and veterans (SMVs) (1). There has been increasing awareness that physical, sensory, cognitive, and/or affective symptoms are often reported many months or years following injury [e.g., (2)] or develop following subconcussive blast exposure (3). Critical for improving patient care is our ability to comprehensively assess the range of problems that individuals with a TBI or blast exposure experience. This goal is complicated by variability in the causes and symptoms associated with these deficits.

Of particular challenge has been assessing TBI- and blast-associated deficits in communication. Hearing loss and tinnitus are among the most prevalent service-connected disabilities for veterans (4) and the incidence of hearing difficulties in service members, particularly those deployed (5), exceeds that of the U.S. working population (6). The monumental increase of blast exposure in deployments has led to an increased incidence of TBI diagnoses and associated auditory impairment (7). Permanent sensorineural hearing loss is reportedly the most prevalent type of auditory impairment in blast trauma, accounting for 35–54% of auditory injury (8). Chandler (9) estimated that 64% of blast-injured service members being treated at a large, U.S. military treatment facility had ongoing hearing loss. Bothersome tinnitus is also often experienced in people with trauma-related injuries, exposure to high levels of occupational noise, and hearing loss; all of which are common in the military population. Approximately 20% of people with chronic tinnitus have bothersome tinnitus that can promote cognitive difficulties, mental health disorders, insomnia, and decreased psychosocial functioning (10–12).

SMVs with a history of TBI and/or blast exposure often report even greater difficulties understanding speech in noisy environments than would be predicted from clinical audiometric assessments, such as pure tone thresholds (13). Clinical tests are often not sensitive enough to quantify speech recognition difficulties (14). In general, individuals with a TBI may appear normal in clinical exams, but suffer in more complex, real-world environments (15).

These findings suggest multiple sources of impairment may occur with TBI or blast exposure that exacerbate speech understanding difficulties in challenging conditions. Though the mechanisms are still under investigation, damage along the peripheral to central auditory pathway may place greater demands on top-down, cognitive systems to compensate, especially in adverse conditions. Individuals with damage to these domain-general systems, as can occur with TBI or blast exposure, may thus be particularly unable to compensate. Indeed, a tight link between auditory and cognitive impairments has been noted in the epidemiological literature (16). Additionally, small-scale studies of civilians have observed associations between auditory and cognitive function in assessing auditory processing abilities (13, 17, 18), though these assessments did not consistently distinguish individuals with and without a history of TBI.

Accurate assessment of communication challenges is critical for mitigating the potential negative social and cognitive consequences of auditory dysfunction including poorer quality of life (19) and job performance and promotion (20). The presence of bothersome tinnitus can have a detrimental impact on a person's emotional, social, mental, and professional life. Tinnitus secondary to blast injury may even be more detrimental due to its sudden emergence instead of gradual onset with progressive sensorineural hearing loss (21).

Complicating the assessment of the impact of TBI and blast exposure on communication is the variability in the causes and symptoms associated with these injuries. For example, mild TBI (mTBI) resulting from blast exposure has been associated with more self-reported hearing difficulty than mTBI resulting from a non-blast mechanism (22). Comorbidities may also cloud our understanding of symptoms of TBI reported years following a TBI. Over 40% of soldiers with mTBI have a comorbid post-traumatic stress disorder (PTSD), and a number of health problems reported by service members with mTBI are strongly influenced by PTSD or depression (23, 24).

Due to increased concern of mental health disorders in the military population independent of auditory status (25, 26), it is imperative that those with bothersome tinnitus and auditory dysfunction are given timely and appropriate treatment options. However, tests of speech recognition in noise and other complex environments as well as tinnitus evaluations are often not part of a standard audiological evaluation.

Given the diversity of factors contributing to challenging speech understanding and hearing and tinnitus problems, we present an initial analysis of a large-scale study of SMVs. This study aims to highlight domains that may be important for comprehensive assessments of the subjective and objective hearing and tinnitus problems of SMVs with or without a history of TBI and/or blast exposure.

## Method

### Participants

SMVs underwent auditory and neuropsychological testing at Walter Reed National Military Medical Center (WRNMMC) as part of the Congressionally mandated 15-Year Longitudinal TBI Study (Sec721 NDAA FY2007) by the Defense and Veterans Brain Injury Center (DVBIC). Details on inclusion criteria, group definition, and recruiting procedures are found in Lange et al. (2). In the current analyses, SMV's first session of complete data was included, yielding 278 participants. Eighteen participants were excluded due to having an equivocal or unknown TBI history. Individuals were also excluded because of invalid cognitive test scores (e.g., performance validity testing) or exaggerated symptom reporting (2, 27) (*n* = 46) or invalid auditory test scores (*n* = 2).

Of the remaining 212 participants, 40% had a history of an uncomplicated mild TBI (mTBI), 29% had greater than an uncomplicated mTBI (i.e., *n* = 16 complicated mTBI, *n* = 14 moderate TBI, *n* = 15 severe TBI, *n* = 16 penetrating TBI), and 31% of had no history of TBI (details in Measures section). 40% of all participants responded on a screening question as having been close enough to an explosive blast to self-report symptoms of a “possible” alteration of consciousness (Blast Exposure question described below). These SMV participants were 93% male and ranged in age from 19.57 to 61.97 years (*M* = 37.69, *SD* = 10.25). Individuals with a history of TBI were tested at least 2.5 months after their date of injury (*M* = 7.33 years, *SD* = 8.15).

### Measures

#### Audiological Screening

Otoscopy was performed to confirm no abnormalities of the tympanic membrane, ear canal, or presence of occluding cerumen. Clear visualization of the tympanic membrane was noted during otoscopy for all participants. Tympanometry measured middle ear function to assess tympanic membrane mobility and compliance and ensured there were no active tympanometric abnormalities. Pure tone air conduction thresholds were measured at octave/interoctave frequencies of 0.25, 0.5, 1, 2, 3, 4, 6, and 8 kHz to determine the degree of hearing loss in each ear. Standard Pure Tone Averages were computed for low frequencies (PTA LF: 0.5, 1, 2 kHz) and high frequencies (PTA HF: 3, 4, 6, 8 kHz) in the better and in the worse ear.

#### Speech Recognition Composite Score

Previous studies have shown that there can be substantial variability in the performance of individuals on different standardized speech tests (28). To obtain a comprehensive estimate of speech-in-noise performance, each participant was tested with five speech-in-noise measures and a composite score was obtained by transforming test scores to have the same polarity (lower scores = better performance), z-transforming, and then averaging. The five measures were: (1) Modified Rhyme Test, (2) Standard and (3) Time-Compressed/Reverberant Quick Speech-in-Noise Test, (4) Listening in Spatialized Noise, and (5) High/Low Context Sentences.

##### Modified rhyme test (MRT)

The MRT (29) is a consonant perception test that requires listeners to identify a monosyllabic word from six alternatives that differ only by the first or last consonant. Each listener completed 40 MRT trials in each ear. Stimuli were masked by speech-shaped noise. Half the trials were presented at a +4 dB SNR and half at a −4 dB SNR. Median response time (MRT RT) was also recorded for each participant.

##### Standard and time compressed/reverberant quick speech-in-noise (QSIN) tests

Each participant completed an adaptive tracking task using IEEE sentences from the Modified QSIN test (30). Separate tracks were used to estimate the 50% speech reception threshold (SRT) for the standard test (diotic speech in four-talker babble) and the speeded-reverb test (time-compressed speech with 4-talker babble at +90 degrees, 4-talker babble at −90°, and a target talker at 0 degrees).

##### Listening in spatialized noise (LISN-S)

In the LISN-S (31), participants repeat target sentences in the presence of two competing talkers who are speaking sentences that could easily be confused with the target speech. Only the high-cue condition of the test was administered, where that target talker was a different sex than the masking talkers and the masking talkers were separated 90 degrees to the left and right of the target. The test estimates the SRT where listeners identify 50% of the words in the target sentences.

##### High/low context sentences (HLCS)

HLCS utilizes the Revised Speech Perception in Noise Test (R-SPIN) sentences (32) to assess comprehension of high and low context sentences in multitalker background noise at a standard or time compressed rate. Participants repeat the entire sentence, which is scored for key words correct to generate a percent correct for each context condition.

#### Subjective Auditory Complaints

##### Tinnitus and hearing survey (THS)

The THS (33) differentiates problems caused by hearing loss from tinnitus or hyperacusis. Participants rate how problematic their hearing or tinnitus has been in myriad situations within the last week (0–4). Hearing and Tinnitus subscores each comprise four questions, with a higher score indicating greater problems.

#### Neurological Symptoms

##### TBI history and severity

TBI severity was non-normally distributed, with greater representation of uncomplicated mTBI. Thus, TBI history (present vs. absent) and TBI severity (no more than uncomplicated mTBI vs. complicated mTBI or more severe) were treated as binary factors. Details of TBI severity categorization are in Lange et al. (2). In sum, TBI severity was classified as: *uncomplicated mTBI* (i) Glasgow Coma Scale (GCS) = 13–15, Post-Traumatic Amnesia (PTA) <24 h, Loss of Consciousness (LOC) <30 min, and/or Alteration of Consciousness (AOC) present, and (ii) no trauma-related intracranial abnormality on CT or MRT; *complicated mTBI* (i) GCS = 13–15, PTA <24 h, LOC <30 min, and/or AOC present, and (ii) trauma-related intracranial abnormality on CT or MRI; *moderate TBI:* LOC 1–24 h, PTA 1–7 days, and ICA present or absent; *severe TBI*: LOC >24 h, PTA >7 days, and ICA present or absent; *penetrating TBI*: breach of the cranial vault and/or dura mater by external object (e.g., bullet, shrapnel) and/or skull fragment (i.e., skull fracture). Individuals with no history of TBI included 41 injured controls (orthopedic/soft tissue injury with no evidence of AOC, LOC, or PTA as result of injury) and 25 non-injured controls.

##### PTSD checklist-civilian version (PCL-C)

The PCL-C is a self-administered questionnaire (34) with 17 items designed to evaluate self-reported PTSD, patterned after the DSM-IV-TR (35) symptom criteria for PTSD. The PCL-C is not limited to military experiences, but open to any traumatic event experienced in their lifetime. Participants rate each item (1–5), with a higher total score indicating greater severity of symptoms.

##### TBI quality of life depression scale (TBIQOL-DEP)

The TBI Quality of Life measurement system (36) assesses self-reported quality of life problems in individuals with a history of TBI. Higher t-scores on the depression scale (TBIQOL-DEP) indicate more severe depressive symptoms.

##### Blast exposure

Participants responded to a question based on the Ohio State University Traumatic Brain Injury Identification Method (37): “*Have you ever been nearby when an explosion or blast occurred, that resulted in you feeling confused, disoriented, or having a loss of memory for a few seconds or minutes (or longer)? Think about any combat-related incidents.”* Participants indicating “yes” were categorized as having been exposed to a blast. While this question screens for self-reported blast exposure, individuals may not have met diagnostic criteria for AOC as revealed through in-depth interviews with the study team.

#### Cognitive Domains

Cognitive function was assessed through neuropsychological testing including components of the Connor's Continuous Performance Test-2 (CPT-II) (38), Wechsler Adult Intelligence Scale-IV (WAIS-IV) (39), Delis-Kaplan Executive Function System (D-KEFS) (40), Trail Making Test (41), and Neuropsychological Assessment Battery [NAB; (42)]. Cognitive domains that have been shown to contribute to speech understanding in adverse conditions [e.g., (43, 44)] were included in analyses. Domain composite scores (27) were calculated by averaging the scaled scores (*M* = 10, *SD* = 3) associated with the following subtests, with higher scores indicating better performance. Tests that do not produce scaled scores were converted prior to calculating the composite domains.

##### Attention and working memory domain

CPT-II Omissions and Hit Reaction Time Standard Error and WAIS-IV Digit Span and Letter-Number Sequencing.

##### Processing speed domain

WAIS-IV Coding and Symbol Search, D-KEFS Color-Word Condition 2, and Trail Making Test Trial A.

##### Executive functioning domain

D-KEFS Verbal Fluency Category Switching and Color Word Interference Test Inhibition, NAB Categories Test, and WAIS-IV Similarities.

##### Verbal fluency domain

D-KEFS Verbal Fluency Letter Fluency and Category Fluency.

### Analyses

One challenge for studies with many predictors is that traditional regression models may be underpowered to test the role of each variable. Building predictive models using stepwise procedures may be subject to over-fitting and sensitivity to multicollinearity, thus limiting generalizability to the population. Here, we employ a penalized regression model (least absolute shrinkage and selection operator, LASSO) that allows for testing large numbers of predictors while minimizing model error and potential for over-fitting (45). LASSO has been used to examine questions about the role of cognitive, sensory, and demographic factors in predicting clinical outcomes for individuals with schizophrenia (46). It has also been used to assess the relative roles of objective and self-reported auditory and cognitive functions in hearing aid outcomes (47).

LASSO beta coefficients for variables that contribute less to the model are forced to be exactly zero via a shrinkage penalty (lambda), allowing for concurrent variable selection and parameter estimation. Only the most contributive variables remain in the final model. The Bayesian version of the LASSO (48) has the added advantage of providing standard errors and a more flexible way of estimating tuning parameters and predictors.

Bayesian LASSO regressions were run in R [version 3.6.0; (49)], using mostly default settings of the blasso function in the package movomvn (version 1.9-13) (50). Models were run with Gibbs sampling, uninformative gaussian priors, and hyperparameters recommended by Park and Casella (48). 10,000 Markov Chain Monte Carlo (MCMC) samples of the model were drawn to achieve stable estimates of predictors and tuning parameters. Beta values represent the median values of the posteriors for each predictor after 1,000 discarded burn-in samples. Resulting estimates of lambda and variance are reported.

Predictors and dependent measures were scaled and centered prior to entering in the model. The predictors for the speech recognition composite model included: age (years), TBI history (0/1), TBI severity (0/1), Blast Exposure (0/1), MRT RT (ms), pure tone average (dB HL) in the better and worse ears for low frequencies (i.e., PTA LF BE, PTA LF WE) and high frequencies (i.e., PTA HF BE, PTA HF WE), PCL-C score, TBIQOL-DEP score, and domain scores for Attention/WM, Processing Speed, Executive Function, and Verbal Fluency. Models for THS Hearing and Tinnitus scores also included the speech composite score as a predictor. Beta estimates indicate the contribution of each variable to the measure of interest, with an associated probability that its contribution is not 0 (contributing factors >0.50).

Variance inflation factors (VIFs) were initially calculated to assess potential multicollinearity across regression predictors before entering them into the models. A VIF > 10 is often used as an indicator of collinearity that could impact model stability. All predictors had a VIF <5.82.

## Results

### Objective Speech Recognition Performance

**Figure 1:**
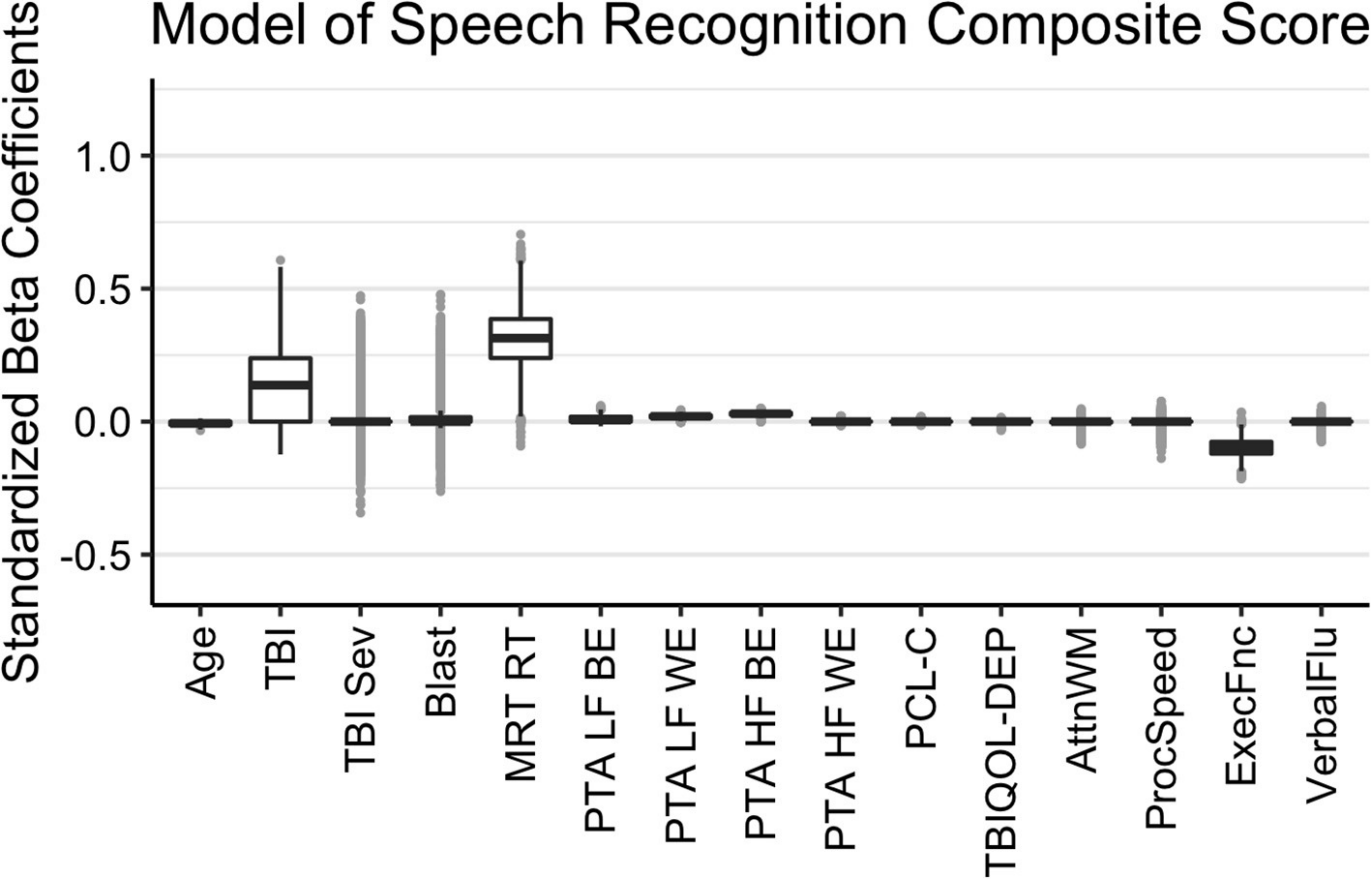
Regression coefficients for the Speech Recognition Composite model. Median intercept (mu) = −0.10 (Q1 = −0.47, Q3 = 0.27).

The results of the Bayesian LASSO predicting speech recognition composite scores are in Table 1 and Figure 1. Factors associated with worse speech recognition performance (higher score) were decreasing age, a history of TBI, slower MRT RT, poorer hearing thresholds for the better and worse ears in the lower frequencies and for the better ear in the high frequencies, and worse executive function. The median variance and lambda penalty parameters were 0.50 and 0.13, respectively. Deviations of the fitted values from the raw values are approximated by an R-square of 0.50.

**Table 1:** Results of Bayesian LASSO regressions.

**Parameter**	**Beta (probability** **≠** **0)**		
	**Speech recog composite**	**THS Hearing**	**THS Tinnitus**
Age	**−0.0069 (0.71)**	0 (0.32)	0 (0.41)
TBI	**0.14 (0.70)**	0 (0.32)	0 (0.50)
TBI severity	0 (0.32)	0 (0.38)	**0.41 (0.96)**
Blast	0 (0.35)	**0.074 (0.62)**	0 (0.41)
MRT response time	**0.020 (0.98)**	0 (0.43)	0 (0.38)
PTA LF BE	**0.0060 (0.62)**	0 (0.36)	0 (0.38)
PTA LF WE	**0.020 (0.97)**	0 (0.49)	0 (0.41)
PTA HF BE	**0.030 (>0.99)**	**0.016 (0.93)**	0 (0.50)
PTA HF WE	0 (0.41)	**0.0078 (0.82)**	0 (0.47)
PCL-C	0 (0.29)	**0.035 (1.00)**	0 (0.37)
TBIQOL-DEP	0 (0.35)	0 (0.51)	0 (0.38)
Attention WM domain	0 (0.34)	0 (0.50)	**−0.0058 (0.60)**
Processing speed domain	0 (0.33)	0 (0.32)	**−0.048 (0.81)**
Executive function domain	**−0.098 (0.98)**	0 (0.40)	0 (0.49)
Verbal fluency domain	0 (0.30)	0 (0.39)	0 (0.38)
Speech recog composite	N/A	0 (0.50)	0 (0.41)

*Beta values represent the median values of the posteriors for each predictor (contributive factors in bold)*.

### Subjective Hearing Difficulties

**Figure 2:**
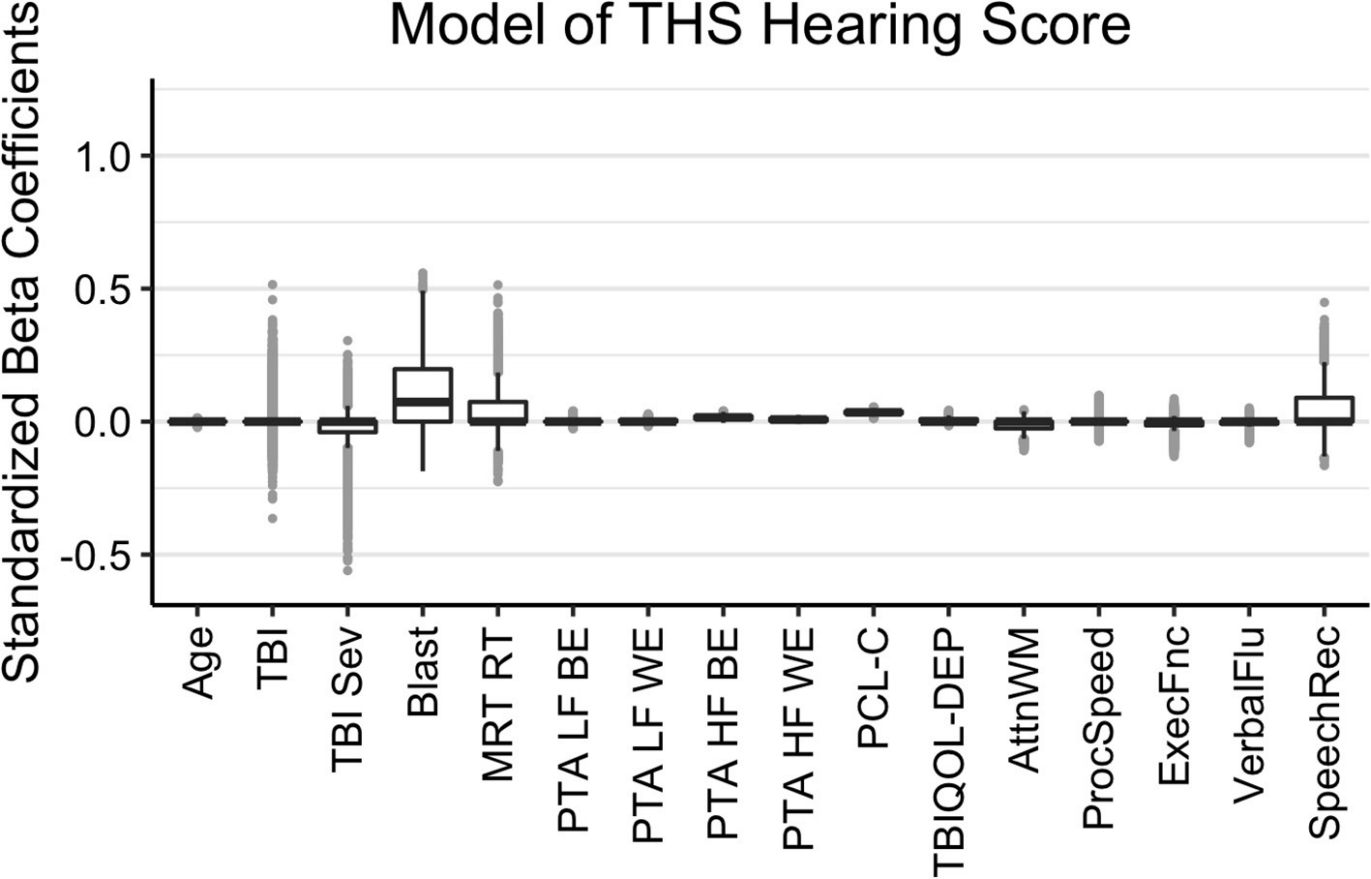
Regression coefficients for self-reported difficulties with hearing (THS Hearing). Median intercept (mu) = −1.53 (Q1 = −1.86, Q3 = −1.21).

The results of the Bayesian LASSO predicting self-reported difficulties with hearing are in Table 1 and Figure 2. Factors associated with more hearing problems (higher THS score) were a history of self-reported blast exposure, poorer hearing thresholds for the better and worse ears in the higher frequencies, and greater reports of symptoms of PTSD. Median variance and lambda penalty parameters were 0.55 and 0.18, respectively. Deviations of the fitted values from the raw values were approximated by an R-square of 0.46.

### Subjective Tinnitus Difficulties

**Figure 3:**
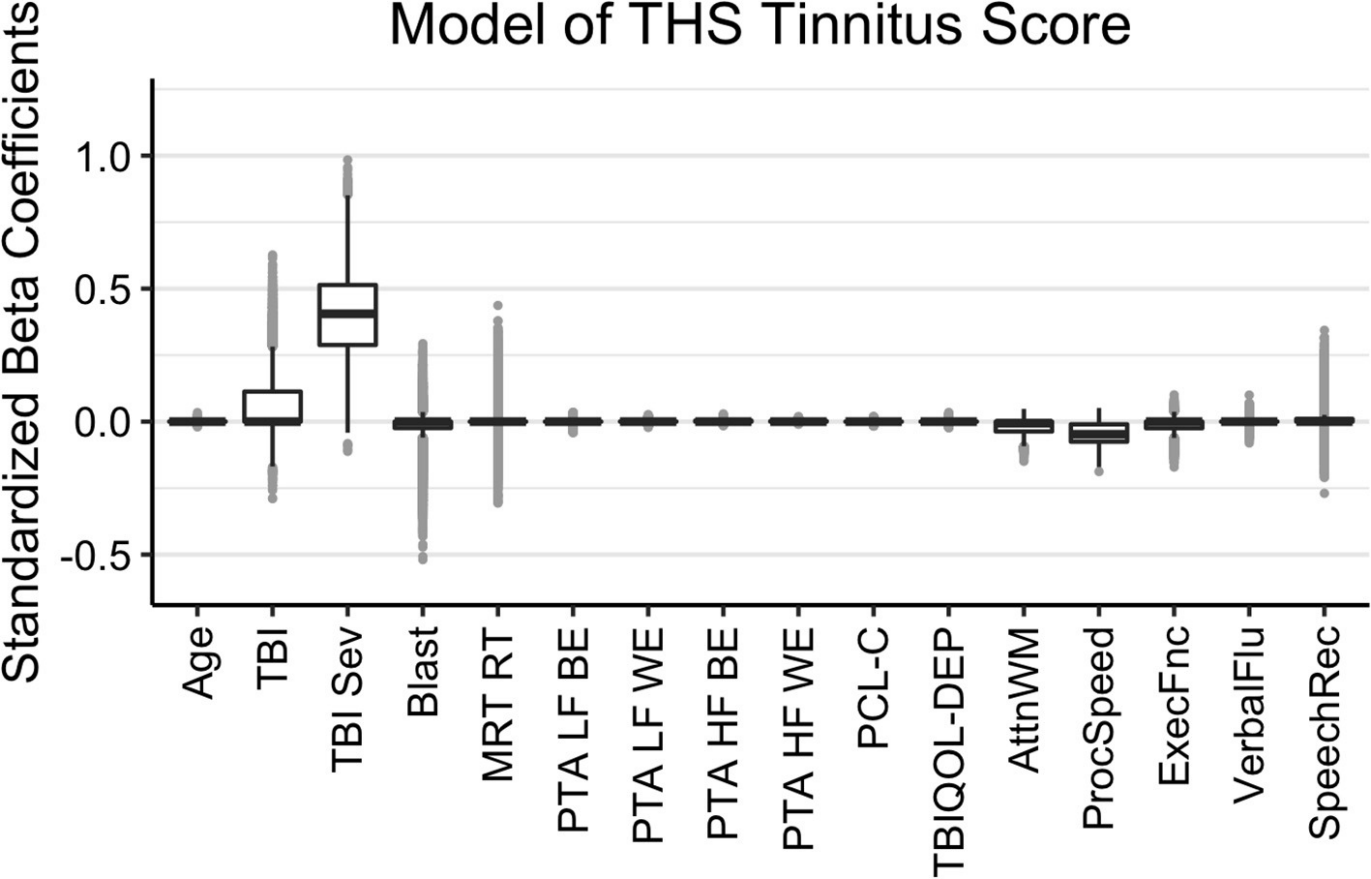
Regression coefficients for self-reported difficulties with tinnitus (THS Tinnitus). Median intercept (mu) = 0.50 (Q1 = 0.14, Q3 = 0.84).

The results of the Bayesian LASSO predicting self-reported difficulties with tinnitus are in Table 1 and Figure 3. Factors associated with more tinnitus problems (higher THS score) were the presence of a more severe TBI, greater reports of symptoms of PTSD, and poorer processing speed. TBI severity was not independent from TBI history due to controls having a value of 0 for both. In this case, LASSO shrinks the less contributive factor to 0. Median variance and lambda penalty parameters were 0.89 and 0.57, respectively. Deviations of the fitted values from the raw values were approximated by an R-square of 0.12.

## Discussion

SMVs with a history of TBI or blast exposure often report difficulties understanding speech in adverse conditions that are difficult to capture with standard audiometric tests. This suggests multiple factors contribute to their speech recognition problems. This study's results are consistent with this: individuals with a self-reported history of blast exposure also reported more severe auditory symptoms than those without, but only those with a history of TBI exhibited significantly worse performance on an objective measure of speech recognition. Furthermore, having a history of a more severe TBI was associated with greater tinnitus complaints (though this model explained little variance overall).

As expected, impairments in pure tone audiometric thresholds contributed to objective and subjective hearing performance. Three of the four thresholds contributed to predicted objective performance. At high frequencies, speech-in-noise performance was dominated by the thresholds in the “better” ear, which reflects that listeners can extract speech information from one ear for binaurally-presented speech. At low frequencies, speech-in-noise performance was primarily determined by thresholds in the “worse” ear. This reflects the important role that low-frequency binaural processing plays in the perception of speech stimuli in complex auditory environments. The performance benefit that listeners get when a noisy signal is spatially separated from a target (i.e., binaural release from masking) depends on the auditory system's ability to compare the amplitude and phases of low-frequency sounds arriving at the two ears. Thus, one would expect performance to be limited by the fidelity of the neural representation of the sound in the worse ear. Previous studies have shown that binaural tasks like auditory localization tend to degrade when the hearing threshold at 500 Hz exceeds 40 dB in the worse ear (51).

MRT RT and executive function also predicted objective speech-in-noise recognition. These results align with previous findings that cognitive factors influence performance on speech-in-noise tests [e.g., (43, 44)]. They also extend the results of small-scale studies of civilians with and without TBI (each total *n* < 35) that observed associations between auditory and cognitive function in assessing speech-in-noise abilities (13, 18).

Surprisingly, an increase, rather than a decrease, in speech-in-noise performance was predicted with aging. This was one of the weaker effects in the model and likely reflects an offset related to another predictor that naturally increases with age, like hearing thresholds. This interpretation is supported by a weak, positive correlation between age and speech composite score [*r*_(210)_ = 0.14, *p* = 0.04] in the overall data when other factors are not partialed out.

In contrast to objective recognition, subjective hearing difficulties were predominantly predicted by thresholds in the better ear at high and low frequencies. Notably, the actual performance level on the objective speech-in-noise tests was not a significant predictor. Responses to THS Hearing questions may have been more likely to be influenced by the audibility of soft sounds than by speech-in-noise understanding. Objective speech-in-noise tests in this study were generally presented at a high enough level to ensure audibility, which could explain why low frequency thresholds in the worse ear contributed more to the objective than the subjective speech measure. Additionally, symptoms of PTSD were related to subjective hearing problems. However, even when including these factors, self-reported blast exposure was still related to hearing complaints. This follows from the finding that mTBIs caused by blast exposure are associated with more self-reported hearing difficulty than those not caused by blast (22). Because blast exposure often accompanies acoustic noise exposure, more detailed blast and noise exposure history information will be needed to better understand this link.

In addition to greater TBI severity, subjective tinnitus complaints were also associated with measures of processing speed and attention/working memory. This finding aligns with research suggesting that bothersome tinnitus coincides with deficits in attention and executive functions (52). However, few factors predicted tinnitus problems in this sample, and the model explained a small amount of variance.

The results of this study highlight that standard audiometric measures may be insufficient to characterize the hearing-related problems of SMVs with a history of TBI or blast exposure. Although the mechanisms are unclear, these data suggest that SMVs with blast exposure or TBI suffer from hearing deficits that go beyond what would be expected from increased hearing thresholds, elevated PTSD and depressive symptoms, and degraded cognitive function that might result from their injuries. Indeed, there was an observable relationship between hearing difficulties and TBI or blast history even when these other factors were included in the models.

One takeaway from this data is that objective speech-in-noise performance, subjective hearing and tinnitus were associated with different injury mechanisms. Objective speech-in-noise performance was best predicted by the presence of any TBI. Subjective hearing was best predicted by a self-reported history of blast exposure and tinnitus was best predicted by the presence of severe TBI. Military audiologists have anecdotally noted that blast-exposed patients tend to report hearing problems that are difficult to validate with clinical tests of speech-in-noise recognition. These data are consistent with those anecdotal observations. However, it is not clear whether increased patient complaints reflect a true performance deficit that was not detected by our speech-in-noise tests or whether it reflects a tendency for blast-exposed listeners to experience greater listening effort even when they achieve the same level of objective performance.

This preliminary, cross-sectional analysis has focused on identifying broad factors that might differentiate the objective hearing performance and subjective hearing complaints of SMVs with TBI from those of SMVs who have not suffered head injuries. As data collection progresses, we hope to be able to identify more specific tests or combinations of tests that might be sensitive to the unique hearing pathologies that exist in this population. The longitudinal nature of the study will also make it possible to track how these hearing problems progress over time and the extent to which they might contribute to the overall quality of life experienced by SMVs with TBI. Perhaps most importantly, we hope to be able to conduct more nuanced analyses to help identify the specific exposures or injury mechanisms that might be responsible for the excess hearing difficulties attributed to blast exposure or TBI in this sample. In the short term, however, these results may serve to highlight the importance of including audiological measures beyond the pure-tone audiogram in studies evaluating chronic effects of TBI. Including cognitive and symptomological assessments may help to better characterize and ultimately better remediate these deficits. However, more work is needed to fully account for the challenges that SMVs with a history of TBI or blast exposure face.

## Data Availability Statement

The datasets presented in this article are not readily available because Department of Defense policy prohibits sharing of sensitive data for this DoD-funded research. Requests to access the datasets should be directed to Stefanie E. Kuchinsky, stefanie.e.kuchinsky.civ@mail.mil.

## Ethics Statement

The studies involving human participants were reviewed and approved by the Institutional Review Board at Walter Reed National Military Medical Center. The patients/participants provided their written informed consent to participate in this study.

## Author Contributions

RL, LF, and TB designed and directed the larger longitudinal project. RL, LF, TB, SL, DB, and ME contributed to the overarching design of this study. DB, SK, and ME conceptualized the specific research question. SK conceptualized and implemented the analysis plan. SK, DB, and ME interpreted the results and wrote the initial draft of the paper. All authors contributed to the article and approved the submitted version.

## Conflict of Interest

The authors declare that the research was conducted in the absence of any commercial or financial relationships that could be construed as a potential conflict of interest.
